# Post-Doc Interviews in the Life Sciences: An Often-Overlooked Process that Is Susceptible to Bias

**DOI:** 10.1093/iob/obz027

**Published:** 2019-10-25

**Authors:** N P Burnett, S A Combes

**Affiliations:** Department of Neurobiology, Physiology and Behavior, University of California, Davis, CA 95616, USA

## Abstract

Post-doctoral training is a critical career stage for researchers in the life sciences, yet interviewing for a post-doctoral position is largely an unregulated process. Without regulation, interviews are susceptible to unconscious biases that may lead to discrimination against certain demographic groups (e.g., women and minorities). Using data from an online survey of post-docs, we show that interview procedures for post-doctoral positions in the life sciences are correlated with several factors (e.g., candidate demographics) in ways that may bias the outcome of interviews. We discuss key components of interviews and suggest that conducting standardized, well-planned interviews that are less susceptible to unconscious biases may help increase the retention of women and under-represented minorities in the life sciences.

## Synopsis

Entrevistas postdoctorales en ciencias de la vida: un proceso que a menudo se pasa por alto y que es susceptible al sesgo (Post-doc interviews in the life sciences: An often-overlooked process that is susceptible to bias) La capacitación posdoctoral es una etapa profesional crítica para los investigadores en ciencias de la vida, pero la entrevista para un puesto postdoctoral es en gran medida un proceso no regulado. Sin regulación, las entrevistas son susceptibles a sesgos inconscientes que pueden conducir a la discriminación contra ciertos grupos demográficos (por ejemplo, mujeres y minorías). Utilizando datos de una encuesta en línea de documentos post-doctorales, mostramos que los procedimientos de entrevista para puestos post-doctorales en ciencias de la vida están correlacionados con varios factores (por ejemplo, los demográficos de los candidatos) en formas que pueden sesgar el resultado de las entrevistas. Discutimos los componentes clave de las entrevistas y sugerimos que realizar entrevistas estandarizadas y bien planificadas que sean menos susceptibles a sesgos inconscientes puede ayudar a aumentar la retención de mujeres y minorías subrepresentadas en las ciencias de la vida. Translated to Spanish by YE Jimenez (yordano_jimenez@brown.edu)

Post-Doc-Vorstellungsgespräche in den Biowissenschaften: Ein oft übersehener Prozess, der anfällig für Verzerrungen ist (Post-doc interviews in the life sciences: An often-overlooked process that is susceptible to bias) Die postdoktorale Ausbildung ist eine kritische Karrierestufe für ForscherInnen in den Biowissenschaften. Vorstellungsgespräche für postdoktorale Stellen sind jedoch weitgehend unreguliert. Ohne Regulierung sind die Gespräche anfällig für unbewusste Vorurteile, die zur Diskriminierung bestimmter demografischer Gruppen (z. B. Frauen und Minderheiten) führen können. Anhand von Daten aus einer Online-Umfrage unter Post-Docs zeigen wir, dass die Vorgehensweisen in Bewerbungsgesprächen für Post-Doc-Positionen in den Biowissenschaften mit verschiedenen Faktoren (z. B. demografischen Merkmalen von KandidatInnen) in einer Weise korrelieren, die das Ergebnis der Gespräche beeinflussen kann. Des Weiteren diskutieren wir Schlüsselkomponenten von Vorstellungsgesprächen. Wir nehmen an, dass die Durchführung standardisierter, gut geplanter Bewerbungsgespräche, die weniger anfällig für unbewusste Voreingenommenheit sind, dazu beitragen kann, den Verbleib von Frauen und unterrepräsentierten Minderheiten in den Biowissenschaften zu erhöhen. Translated to German by F Klimm (frederike.klimm@biologie.uni-freiburg.de)

In the life sciences, barriers within and especially between career stages (e.g., graduate student to post-doctoral researcher to faculty) have contributed to the low retention and under-representation of women and racial and ethnic minorities at later career stages (Nelson 2007; Allen-Ramdial and Campbell 2014; El-Alayli et al. 2018; Rangel et al. 2018; Witteman et al. 2019). Unfair hiring practices are one prominent barrier faced by members of under-represented groups (Mavriplis et al. 2010; Sheltzer and Smith 2014; Sensoy and DiAngelo 2017), partly due to unconscious biases that interviewers may possess against individuals from these groups (Derous et al. 2016, 2017). In contrast to interviews for graduate school admission and faculty positions, interview procedures for post-doctoral (post-doc) positions are often established by the principal investigators (PIs) rather than the institutions. Without regulatory oversight by institutions, PIs may rely on informal or non-standardized interviewing procedures, increasing the risk that unconscious biases are introduced into interviews (Sheltzer and Smith 2014; Derous et al. 2016). Because post-doc training is critical to the long-term career trajectories of scientists (Su 2013), and because biased interviews could limit the access that certain demographic groups have to post-doc positions (Derous et al. 2016), we designed an online survey to test the hypothesis that interview procedures for post-doc positions were linked to the demographic characteristics of the candidates (gender, race, and ethnicity), the format of interviews (in-person or electronic), the presence of a previous relationship with the PI (did or did not know the PI before the interview), and the funding source for the position (self-funded, e.g., by a fellowship to the candidate, or funded by other sources).

## Survey questions

We asked post-docs currently working in the life sciences about the interviews they experienced when applying to their current positions. An interview was defined as “conversations that occurred between you and your PI (or hiring committee) that were used to evaluate your suitability as a post-doctoral fellow in the PI’s laboratory or group” and we specified that interviews occurred “between when you initially applied to or inquired about the post-doctoral position and when you were officially offered the position.” For demographic data, we asked each post-doc’s gender, race, and ethnicity. We also asked whether each post-doc identified with the same gender as the PI. To characterize the nature of the post-doc position and interview, we asked whether the post-doc was formally interviewed (yes or no), whether they previously knew the PI (yes or no), what type of funding paid their salary (self-funded, e.g., through a post-doctoral fellowship, or funded by other sources), and the interview’s medium (electronic, e.g., by Skype, or in-person).

We then asked each post-doc to classify their interview in four ways, two of which dealt with logistics (interview duration and activities) and two of which dealt with the post-doc’s perceptions of the interview (interview content and structure). In cases where we provided multiple possible responses or asked post-docs to rate their experience along a numerical scale, we coded these as binary responses for logistic regression based on the distribution of responses (Supplementary Table S1). To classify interview duration, post-docs were asked how long their interview lasted (including breaks, overnight stays, etc.), and responses were coded as “< 1 hour” or “> 1 hour” (grouping all options from 1 to 2 h through multiple days together; Supplementary Table S1). To examine interview activities, we asked post-docs whether they gave a presentation or demonstrated any technical skills as part of their interview (yes or no). To classify interview content, we asked post-docs to rank the content discussed during their interview along a numerical scale from entirely work-related to not at all work-related, and responses were coded as “mostly work-related” or “not mostly work-related” (Supplementary Table S1). Finally, to classify interview structure, we asked post-docs to rank how structured their interview was along a numerical scale from highly structured (pre-determined questions or exercises) to highly open-ended (unregimented conversation), and responses were coded as “mostly structured” or “mostly unstructured” (Supplementary Table S1).

## Survey distribution and analysis

Between June and November 2018, we sent the survey to 2191 post-docs working in the life sciences at universities in the United States (collecting e-mail addresses from institution websites) and also sent the survey to 171 administrators to distribute to post-docs at their institutions. We restricted our analyses to the 342 post-docs who completed the survey and acknowledged having an interview for their position (Supplementary Table S2). The gender of respondents was either female or male, and we pooled post-docs identifying with races or ethnicities that are statistical minorities in the United States into a single “minority” group, which included people who identified as Asian, Black or African American, American Indian or Alaskan Native, Hispanic or Latinx, or multiple races and/or ethnicities. The demographic composition of our respondents was similar to that of people who earned doctorates in the life sciences in 2017, the most recent year for which data is available: 55% of earned doctorates were female, 45% were male, 28% were minorities, and 68% were white (National Science Foundation, National Center for Science and Engineering Statistics 2018). Among our survey respondents, 56% were female, 44% male, 34% were minorities, and 66% were white. Respondents identifying as Asian were the most common sub-group within the minority category (52% of females, 72% of males). Although we combined multiple racial and ethnic groups here for statistical purposes, it is important to note that different demographic groups can experience unique challenges in interviews (e.g., Derous et al. 2016, 2017).

We used a logistic regression model to test for correlations between responses to each question (interview duration, activities, content, and structure) and the demographic characteristics of the post-docs, or other characteristics of the interview (previous relationship with PI, funding type, etc.; Peng et al. 2002). The regression model was of the form:

Response ∼ Gender * Minority Status + PI Relationship + Interview Medium + Funding

with two levels for each factor, and allowing for an interaction between the two demographic characteristics of the post-doc. We initially also included a term for whether the PI and the post-doc identified as the same gender, but preliminary analyses showed that this did not significantly improve the model’s predictive power for any response variable (Likelihood Ratio Tests, *P* > 0.05), so this term was dropped from the final model. All statistical analyses were done in R Statistical Software (www.R-project.org). Factors were evaluated with the “wald.test” function in the aod package (Lesnoff and Lancelot 2012), pairwise comparisons of factor levels were computed with the “lsmeans” function in the lsmeans package (Lenth 2016), and results are shown in the corresponding figures. Full outputs of the models are given in Supplementary Tables S4–S7.

## Survey results

**Fig. 1:**
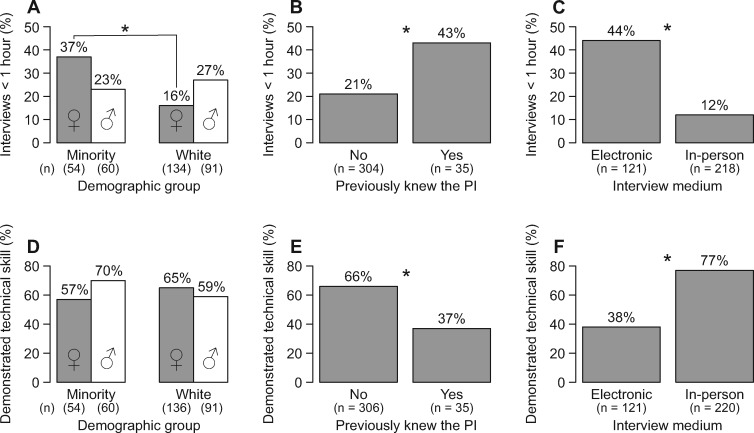
Statistically significant predictors of interview logistics, based on results of logistic regression analyses (Supplementary Tables S4–S5). (**A**–**C**) The percent of interviews that were <1 h long, broken down by (A) demographic group (female = ♀, male = ♂), (B) whether the post-doc previously knew the PI, and (C) the interview’s medium. (**D**–**F**) show the percent of interviews in which the post-doc demonstrated a technical skill, broken down by (A) demographic group, (B) whether the post-doc previously knew the PI, and (C) the interview’s medium. Asterisks show significantly different pairwise comparisons (*P *<* *0.05) with Bonferroni *P*-value adjustments for multiple comparisons. Total number of respondents, *n*, for each demographic group is given in parentheses below the respective bar.

We found that the duration of post-doc interviews was correlated with the post-doc’s demographics, their previous relationship with the PI, and the interview’s medium (Fig. 1). A post-doc’s demographics (gender × minority status) were a strong predictor of interview duration (*P *=* *0.005; Supplementary Table S4), with a significantly greater percentage of minority females having short interviews (< 1 h) than white females (Fig. 1A). Demographics were also a strong predictor of whether post-docs gave a presentation or demonstrated a technical skill during their interview (*P *=* *0.048; Supplementary Table S5), with minority males generally doing so more than members of other groups (Fig. 1D). If a post-doc had a previous relationship with the PI or if the interview was conducted electronically rather than in-person, the interview was more likely to be < 1 h (*P *<* *0.005; Supplementary Table S4, Fig. 1B and C), and the post-doc was less likely to give a presentation or demonstrate a technical skill (*P *<* *0.005; Supplementary Table S5; Fig. 1E and F).

**Fig. 2:**
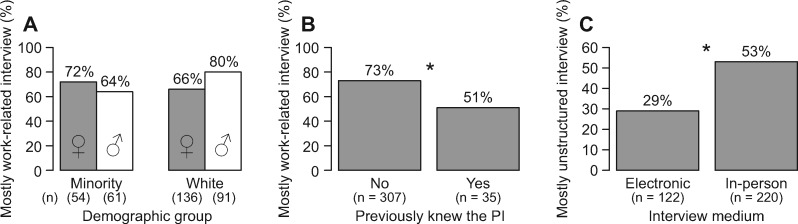
Statistically significant predictors of an interview’s content and structure, based on results of logistic regression analyses (Supplementary Tables S6–S7). (**A** and **B**) The percent of interviews that were perceived as being mostly work-related, broken down by (A) demographic group (female = ♀, male = ♂) and (B) whether the post-doc previously knew the PI. (**C**) The percent of interviews that were perceived as being mostly unstructured, broken down by the interview’s medium. Asterisks show significantly different pairwise comparisons (*P *<* *0.05) with Bonferroni *P*-value adjustments for multiple comparisons. Total number of respondents, *n*, for each demographic group is given in parentheses below the respective bar.

We also found that the content and structure of interviews were correlated with some of the model’s factors (Fig. 2). A post-doc’s demographics (gender × minority status) were correlated with the interview’s content (*P *=* *0.026; Supplementary Table S6), with more white males experiencing interviews that were mostly work-related (Fig. 2A). Post-docs who did not previously know the PI were also more likely to have interviews that were mostly work-related (*P *=* *0.026; Supplementary Table S6, Fig. 2B). Interviews that were conducted electronically rather than in-person tended to be more structured (*P *<* *0.005; Supplementary Table S7), with approximately half of the in-person interviews being perceived by the post-doc as mostly unstructured (Fig. 2C).

Lastly, we re-analyzed the dataset without the survey data from respondents identifying as Asian, to determine whether these data (comprising 72 of the 115 respondents within the minority group) were the sole driver of our results. The trends for each response variable in the reduced dataset were similar to those found with the full dataset but with a smaller effect, likely due to the low sample size of the minority group with Asian respondents excluded (*n *=* *43). Similarly, the low sample size for all minority respondents (*n *=* *115; as compared to white respondents, *n *=* *227) within our full dataset may have prevented us from detecting large effects of demographics for interview components like content and structure, while low sample sizes for respondents who previously knew their PI (*n *=* *35) may have influenced the model’s interpretation of that factor.

## Why is interview style important?

The logistics, content, and structure of an interview can affect its outcome in many ways, some of which are counterintuitive. Long interviews may be interpreted as unfair to candidates, but increasing an interview’s duration can help counteract unconscious biases held by the interviewer (Derous et al. 2016). Thus, the longer interviews reported by white females in our study may have helped to counteract unconscious biases held by their PIs, whereas the shorter interviews reported by minority females may not have provided this opportunity (Fig. 1A). Non-work-related content may also seem unprofessional and potentially biased, but discussing personal, non-work matters is useful for establishing rapport with candidates, helping them to relax, and assessing their inter-personal skills and personalities (Huffcutt et al. 2001; Barrick et al. 2010). Additionally, an interview’s structure is important for minimizing interviewers’ biases (Levashina et al. 2014). Well-structured interviews ensure that candidates receive the same questions or exercises, and they can include different segments—like an initial rapport-building segment (e.g., discussing hobbies) followed by a question-and-answer segment—that are designed to help the interviewer combat their own assumptions and misconceptions about the candidates (Derous et al. 2016).

The circumstances of the interviews (e.g., knowing the PI before the interview and whether interviews were electronic or in-person) were also strongly correlated with interview logistics, content, and structure. While our study did not necessarily compare candidates interviewing for the same position or with the same PI, our results show that interviews are likely to be biased by these external factors.

Finally, it is important to note that our survey considered only successful interviews that resulted in a post-doctoral position, and thus we are not able to pinpoint the mechanisms driving these statistical trends. For instance, we still cannot say whether post-docs who do not know the PI before their interviews are generally offered longer interviews that are mostly work-related, or whether these candidates are more often successful at receiving offers when their interviews are longer and mostly work-related—as opposed to candidates who already know the PI and may receive offers even with the shorter, less work-related interviews. However, the differences in interview logistics, content, and structure that we report here suggest that biases are being introduced into post-doc interviews (e.g., shorter interviews for minority females than for white females, Fig. 1A). While these differences may be due to improvised, unstructured interview procedures, they may also be due to structured, but poorly designed interviews that do not counteract the PIs’ biases (e.g., short interviews with few rapport-building questions, in-person interviews for only a portion of the applicant pool).

Of course, unconscious biases are not the only biases that can influence an interview. A candidate can face discrimination based on explicit biases against their race, ethnicity, gender, and/or reproductive status, among other factors (Trix and Psenka 2003; Sheltzer and Smith 2014; Derous et al. 2017; Rangel et al. 2018). Candidates who identify with multiple stigmatized groups, such as minority females, may experience unique types of discrimination (Crenshaw 1989), such as the shorter interviews experienced by minority females in our study (Fig. 1A). Biases can also affect other parts of the hiring process; for example, letters of recommendation written for women often contain language that is more critical and raises more doubts about the candidate than letters written for men (Trix and Psenka 2003; Madera et al. 2019). Addressing biases at the individual, cultural, and institutional levels (Timmers et al. 2010; Applebaum 2019) is necessary to eliminate biases that affect important career transitions in science. Thus, to counteract potential biases in post-doc interviews, it may be necessary for PIs to more carefully design their interviews, and also for institutions to provide some regulations for post-doc interview procedures. Together, these changes could lead to increased retention of under-represented groups at later career stages in the life sciences.

## Recommendations

Potential bias in post-doc interviews can be reduced by planning interviews of sufficient duration, with a standardized structure that contains the same content for all candidates. Standardizing the structure and content of interviews between different candidates can help PIs normalize the way in which they evaluate candidates (e.g., comparing candidates from stigmatized and non-stigmatized backgrounds by the same criteria), and this can help counteract a PI’s own initial biases, assumptions, and misconceptions (Levashina et al. 2014; Derous et al. 2016). We also found that the circumstances of an interview (e.g., medium of the interview and whether the candidate previously knew the PI) were correlated with its content and structure. Some candidates may be unable to travel for in-person interviews or may feel stigmatized during an in-person interview (Straus et al. 2001), so PIs should plan ahead and coordinate with candidates so that all interviews are held under equitable conditions.

### Recommendations for conducting equitable post-doctoral interviews

- Longer interviews give the PI more time to overcome any unconscious biases about candidates from stigmatized groups, so we recommend that interviews should be as long as reasonably possible. Interviews should be of the same duration for each candidate, giving the PI a similar amount of exposure to all applicants.
- Interviews should all have the same pre-determined structure, which can include rapport-building periods (e.g., casual but directed conversations), activities (e.g., presentations or other demonstrations of technical skills), and work-related question-and-answer sections.
- PIs should plan ahead and coordinate with candidates so that all interviews are conducted using the same medium (e.g., video, phone call, or in-person)

Our recommendations cover only a handful of the components that can shape an interview and influence its outcome. PIs who are committed to reducing and counteracting biases that affect interviews should actively search for the most recent research about known sources of bias in interviews and modify their own practices accordingly. We hope this article motivates members of the scientific community to examine their own interview practices and begin conducting standardized and well-structured interviews within their own research groups, and perhaps even to petition their home institutions to create guidelines or requirements for conducting equitable post-doc interviews.

## Supplementary Material

obz027_Supplementary_Data
