# Supplementary material for: Post-Doc Interviews in the Life Sciences: An Often-Overlooked Process that Is Susceptible to Bias
Source: Integr Org Biol. 2019 Oct 25;1(1):obz027. doi: 10.1093/iob/obz027 (PMC7671148; doi:10.1093/iob/obz027)
Supplement: obz027_Supplementary_Data [file obz027_supplementary_data.docx]

Post-doc interviews in the life sciences: An often-overlooked process that is susceptible to bias

Nicholas P. Burnett, Stacey A. Combes

**Supplementary Material**

| Survey question | Possible responses & coding for logistic regression | | | | | |
| --- | --- | --- | --- | --- | --- | --- |
| **How long was your interview?** (including breaks, overnight stays, etc.) | < 1 hour | 1 - 2 hours | 2 - 6 hours | 6 hours - 1 day | 1 day | multiple days |
| *Interview duration* coded as: | < 1 hour | > 1 hour | | | | |
|  | | | | | | |
| **How would you rank the content that was discussed during your interview**? | 1 (entirely work-related) | 2 | 3 | 4 | 5 (not at all work-related) | |
| *Interview content* coded as: | mostly work-related | | not mostly work-related | | | |
|  | | | | | | |
| **How would you rank your interview’s structure?** | 1 (highly structured | 2 | 3 | 4 | 5 (highly open-ended) | |
| *Interview structure* coded as: | mostly structured | | | mostly unstructured | | |

**Table S1:** Coding of multiple-choice survey questions for logistic regression

|  | **N** | **Same gender as PI** | **Previously knew PI** | **Interviewed in-person** | **Self-funded** | **Interview < 1 hour** | **Demonstrated skill** | **Mostly work-related** | **Mostly structured** |
| --- | --- | --- | --- | --- | --- | --- | --- | --- | --- |
| **Female** |  |  |  |  |  |  |  |  |  |
| Minority | 54 | 22 | 5 | 33 | 4 | 34 | 31 | 39 | 31 |
| White | 136 | 44 | 14 | 99 | 32 | 113 | 88 | 90 | 72 |
| **Male** |  |  |  |  |  |  |  |  |  |
| Minority | 61 | 29 | 6 | 29 | 11 | 46 | 42 | 39 | 38 |
| White | 91 | 57 | 10 | 59 | 19 | 66 | 54 | 73 | 49 |

**Table S2.** Total sample size for each demographic group, and number of responses for one of the two possible answers of each survey question.

|  | **Numerical values used for factor levels in model** | |
| --- | --- | --- |
| **Factors** | **0** | **1** |
| Gender | Female | Male |
| Minority status | Minority | White |
| Previously knew the PI | No | Yes |
| Interview medium | Electronic | In-person |
| Funding | Externally-funded | Self-funded |
|  | **Numerical values used for response levels in model** | |
| **Response Variable** | **0** | **1** |
| Interview duration | < 1 hour | > 1 hour |
| Interview activities | No demonstration | Demonstration |
| Interview content | Not mostly work-related | Mostly work-related |
| Interview structure | Not well-structured | Well-structured |

**Table S3.** Key to the numerical values that were assigned to each factor level in the model. These values can be used to interpret the logistic regression model output.

| **Term** | **β** | **S.E. (β)** | **Wald’s *X*^2^** | **df** | ***P*-value** |  |
| --- | --- | --- | --- | --- | --- | --- |
| Intercept | -0.41 | 0.35 | 1.4 | 1 | 0.235 |  |
| Gender | 1.01 | 0.47 | 4.6 | 1 | 0.031 | * |
| Minority status | 1.10 | 0.41 | 7.3 | 1 | 0.007 | * |
| Relationship with PI | -1.56 | 0.42 | 13.6 | 1 | < 0.005 | * |
| Interview medium | 1.86 | 0.31 | 36.1 | 1 | < 0.005 | * |
| Funding | 0.74 | 0.48 | 2.4 | 1 | 0.121 |  |
| Gender*Minority status | -1.67 | 0.60 | 7.8 | 1 | 0.005 | * |

**Table S4.** Results for logistic regression of interview duration (< 1 hour *vs.* > 1 hour) as a function of gender, minority status, previous relationship with PI, the interview’s medium, and the position’s funding.

| **Term** | **β** | **S.E. (β)** | **Wald’s *X*^2^** | **df** | ***P*-value** |  |
| --- | --- | --- | --- | --- | --- | --- |
| Intercept | -0.72 | 0.35 | 4.4 | 1 | 0.037 | * |
| Gender | 1.01 | 0.45 | 5.0 | 1 | 0.026 | * |
| Minority status | 0.14 | 0.38 | 0.1 | 1 | 0.715 |  |
| Relationship with PI | -1.71 | 0.40 | 17.9 | 1 | < 0.005 | * |
| Interview medium | 2.04 | 0.28 | 52.8 | 1 | < 0.005 | * |
| Funding | -0.09 | 0.34 | 0.1 | 1 | 0.786 |  |
| Gender*Minority status | -1.08 | 0.54 | 3.9 | 1 | 0.048 | * |

**Table S5.** Results for logistic regression of interview activities (demonstrating technical skill *vs.* not) as a function of gender, minority status, previous relationship with PI, the interview’s medium, and the position’s funding.

| **Term** | **β** | **S.E. (β)** | **Wald’s *X*^2^** | **df** | ***P*-value** |  |
| --- | --- | --- | --- | --- | --- | --- |
| Intercept | 1.20 | 0.35 | 11.6 | 1 | < 0.005 | * |
| Gender | -0.43 | 0.41 | 1.1 | 1 | 0.300 |  |
| Minority status | -0.27 | 0.36 | 0.5 | 1 | 0.462 |  |
| Relationship with PI | -0.92 | 0.37 | 6.2 | 1 | 0.013 | * |
| Interview medium | -0.24 | 0.27 | 0.8 | 1 | 0.370 |  |
| Funding | 0.08 | 0.32 | 0.1 | 1 | 0.797 |  |
| Gender*Minority status | 1.17 | 0.52 | 5.0 | 1 | 0.026 | * |

**Table S6.** Results for logistic regression of interview content (mostly work-related *vs.* not) as a function of gender, minority status, previous relationship with PI, the interview’s medium, and the position’s funding.

| **Term** | **β** | **S.E. (β)** | **Wald’s *X*^2^** | **df** | ***P*-value** |  |
| --- | --- | --- | --- | --- | --- | --- |
| Intercept | -0.93 | 0.33 | 8.1 | 1 | 0.005 | * |
| Gender | -0.15 | 0.40 | 0.1 | 1 | 0.711 |  |
| Minority status | 0.05 | 0.34 | < 0.1 | 1 | 0.886 |  |
| Relationship with PI | 0.21 | 0.37 | 0.3 | 1 | 0.570 |  |
| Interview medium | 0.95 | 0.25 | 14.5 | 1 | < 0.005 | * |
| Funding | 0.08 | 0.29 | 0.1 | 1 | 0.779 |  |
| Gender*Minority status | 0.20 | 0.48 | 0.2 | 1 | 0.680 |  |

**Table S7.** Results for logistic regression of interview structure (mostly unstructured *vs.* not) as a function of gender, minority status, previous relationship with PI, the interview’s medium, and the position’s funding.
